# The association between attention‐deficit/hyperactivity disorder and narrative language: What is the role of executive function?

**DOI:** 10.1002/jcv2.70007

**Published:** 2025-02-25

**Authors:** Ida Bonnerup Jepsen, Cecilia Brynskov, Per Hove Thomsen, Charlotte Ulrikka Rask, Rikke Lambek

**Affiliations:** ^1^ Department of Psychology and Behavioural Sciences Aarhus University Aarhus Denmark; ^2^ Department of Nordic Studies and Linguistics University of Copenhagen Copenhagen Denmark; ^3^ Research Unit Department of Child and Adolescent Psychiatry Department of Clinical Medicine Aarhus University Hospital Psychiatry Aarhus University Aarhus Denmark

**Keywords:** ADHD, executive function, language, narrative, storytelling

## Abstract

**Background:**

Research suggests that Attention‐Deficit/Hyperactivity Disorder (ADHD) may be associated with narrative language (or storytelling) difficulties, and executive functioning is hypothesized to underlie this association. However, the contribution of executive function to the narrative language production of children with ADHD is unclear and understudied. Accordingly, this study examined the association between ADHD and narrative language and whether it was mediated by executive function.

**Methods:**

Children with a clinical diagnosis of ADHD (*n* = 46) and a comparison group of neurotypical children (*n* = 40) aged 7–11 years completed a narrative language task, as well as laboratory measures of executive function (i.e., working memory updating and response inhibition) and core language.

**Results:**

Children with ADHD produced narratives with more ambiguous references (*β* = 0.72, *p <* 0.001), less overall coherency (*β* = −0.59*, p* = 0.001), and more morpho‐syntactic errors (*β* = 0.47, *p* < 0.05). There was a significant indirect effect of core language on the association between ADHD and morpho‐syntactic errors (*β =* 0.25, *p* < 0.05). No other indirect effects were statistically significant.

**Conclusions:**

While ADHD was associated with several central aspects of narrative language production, executive function did not mediate this association. Clinicians working with children with ADHD should be aware of the potential presence of co‐occurring narrative language difficulties and that narrative language support may need to target language (e.g., grammar).

**Key Points**

- An association between Attention‐Deficit/Hyperactivity Disorder (ADHD) and narrative language difficulties has previously been suggested and is hypothesized to result from executive function deficits.
- In the current study, ADHD was associated with producing narratives that were less coherent overall and contained more ambiguous references and morpho‐syntactic errors compared to NCs. Executive function did not explain this association.
- Clinicians working with children with ADHD should be aware of the possible presence of narrative language difficulties and their impact on children's functioning.
- Children with ADHD and co‐occurring narrative language difficulties may benefit from language‐targeted support to improve structural aspects of storytelling (such as morpho‐syntactic errors) while more research is required to understand which factors affect narrative language in children with ADHD.

## INTRODUCTION

Attention‐Deficit/Hyperactivity Disorder (ADHD) is diagnosed based on a persistent pattern of inattention and hyperactivity/impulsivity that interferes with functioning across multiple domains (e.g., in school and at home; American Psychiatric Association, 2013). Increasing evidence suggests that ADHD is associated with language difficulties (Tannock, 2018). For instance, children with ADHD have a higher risk of developing language disorders (Sciberras et al., 2014), have a higher rate of pragmatic language difficulties (Carruthers et al., 2021), and obtain lower scores on standardized language tasks (Korrel et al., 2017) than comparison children. However, language difficulties in children with ADHD are often overlooked, and even when they are identified, targeted language support is seldom provided (Chan & Fugard, 2018; Sciberras et al., 2014). While it is unclear why this is the case, it has been suggested that the salience of ADHD symptoms as well as urgency for the child and family to receive support for ADHD may overshadow co‐occurring language difficulties (Tannock, 2018). Given the central role of language in development, understanding the association between ADHD and language is crucial.

Narrative language refers to the ability to understand and produce stories (Berman et al., 1994). It is a form of discourse (Boudreau, 2008), and an integral part of social communication (Carruthers et al., 2021). During development, narrative language becomes increasingly coherent and complex (Berman et al., 1994), and narrative language proficiency is considered crucial to successful social and academic functioning (Boudreau, 2008). For instance, in neurotypically developing children, proficiency in narrative language, in particular production of coherent stories, is associated with social competency (Capps et al., 2000; Norbury & Bishop, 2003) and as well as higher scores on academic achievement tests, reading comprehension, and math (Boudreau, 2008). As a result, difficulties with narrative language could negatively affect several areas of development and functioning. A recent meta‐analysis (Jepsen et al., 2022) found that children with ADHD produced narrative language that was less coherent (Hedges' *g* = 0.58), contained more ambiguous references (Hedges' *g* = 0.52), more disruptive errors (Hedges' *g* = 0.41), and had less syntactic complexity (Hedges' *g* = 0.39) than comparison children. Overall, this suggests that many children with ADHD may have difficulties with narrative language and especially with coherency and structural aspects of language, which may negatively impact their everyday communication skills. While lending preliminary support to an association between narrative language and ADHD, the meta‐analysis also concluded that there is a need for more studies and with larger sample sizes (e.g., 11 of 16 studies had fewer than 30 participants with ADHD; Jepsen et al., 2022). Additionally, several relevant narrative aspects remain understudied (e.g., only six studies investigated syntactic complexity), and the majority of studies did not adjust for relevant factors, such as age and sex (Jepsen et al., 2022). Consequently, more research is needed to examine the robustness of the association between ADHD and narrative language.

It is currently unclear why narrative language difficulties may co‐occur with ADHD (Jepsen et al., 2022; Kuijper et al., 2017). The most widely proposed hypothesis is that executive function deficits, particularly working memory updating (WMU; the ability to monitor, code, and replace incoming information according to the relevance of the task at hand; Miyake et al., 2000) and response inhibition (IN; the ability to deliberately inhibit a dominant, automatic, or prepotent response; Miyake et al., 2000), influence the ability to organize and plan speech, resulting in more errors and less coherency (Barkley, 1997; Tannock & Schachar, 1996; Westby & Watson, 2021). To date, only a handful of studies have investigated the association between narrative language and executive function in children with ADHD—using varying narrative task procedures and producing mixed results (Flory et al., 2006; Koltun, 2004; Kuijper et al., 2015, 2017; Parigger, 2012). One study found that WMU, but not IN, was associated with correct referencing and syntactic complexity (Kuijper et al., 2017), while another (based on the same sample, but using a different narrative task) found WMU and IN to be associated with correct referencing (Kuijper et al., 2015). Other studies investigating executive functions such as WMU, IN, and/or shifting have not found them to be significantly associated with any aspect of narrative language (Flory et al., 2006; Koltun, 2004; Parigger, 2012). In sum, previous studies have varied with respect to which narrative task they have used as well as which narrative aspects they have examined. Furthermore, when executive function has been examined, only one task has been applied to do so. As the use of more than one task is typically recommended when assessing a given executive function domain (e.g., Snyder et al., 2015), this hinders robust conclusions. Given the scarcity of studies, conflicting results, and methodologic issues, more research is needed to determine the role of executive function in relation to narrative language in children with ADHD.

While executive functioning is hypothesized to be the main contributor to narrative language production in ADHD (see e.g., Tannock & Schachar, 1996), core language skills (i.e., general proficiency in structural language; Tannock, 2018; Semel et al., 2003) have also been suggested to contribute, such that poorer language skills lead to more incoherent narratives in children with ADHD (Carruthers et al., 2021). While understudied, a handful of studies have found that differences with respect to coherence (Freer et al., 2011; Lorch et al., 2010) or syntactic complexity (Baixauli Fortea et al., 2018; Rumpf et al., 2012) between children with ADHD and comparison children disappear when controlling for performance on standardized language tasks. However, other studies have found the narrative incoherency of children with ADHD to persist even after controlling for core language (Baixauli Fortea et al., 2018; Staikova et al., 2013). As the evidence is mixed, studies are few, and they have generally only examined one aspect of core language (e.g., vocabulary), and in relation to select narrative aspects, it is currently unclear whether core language abilities contribute to narrative language difficulties in children with ADHD. Investigating what contributes to the association between ADHD and narrative language difficulties is important as it determines what is targeted in an intervention.

Overall, more research is needed to understand the association between ADHD and narrative language, including the role of executive function and core language. Consequently, the current study had two major aims. The first aim was to build on the previous meta‐analysis by Jepsen et al. (2022) by investigating the association between ADHD and the aspects of narrative language production where the meta‐analysis had identified significant group differences (i.e., coherence, cohesion, disruptions, syntactic complexity, and Internal state language (ISL)). Based on the meta‐analysis, we expected to find that having ADHD was associated with all narrative aspects, and in particular, aspects related to coherency of speech (i.e., coherence, ambiguous references, and disruptive errors). The second aim was to investigate whether any association between ADHD and narrative language was mediated by executive function (WMU and IN) and/or core language. We investigated WMU and IN, as these executive function domains have previously been theoretically and empirically associated with narrative language (Kuijper et al., 2015, 2017; Westby & Watson, 2021). Based on the leading hypothesis in the field, it is possible that WMU and IN mediate the association between ADHD and narrative language. However, based on previous research, it is also possible that this is the case for only one of these functions, or for neither of them. Due to conflicting previous results, no specific hypothesis was formulated concerning the role of core language.

## METHOD

### Participants

All children (*n* = 746) between the ages of 7 and 11 consecutively referred over a 2.5‐year period to a specialist ADHD clinic in the Danish Child and Adolescent Mental Health Services were invited to participate in the study. A comparison group of neurotypical children (NC; *n* = n/a) were recruited from 11 schools located in the same geographical area as the clinic. A total of 56 children with possible ADHD and 40 NCs (including 4 sib‐pairs) agreed to participate. To be eligible for inclusion in the study, the children had to be between the ages of 7 and 11 years, 11 months and 30 days, be born after 32 weeks (birth weight ≥ 1500 gr.), have Danish as their first language, as well as an IQ ≥ 70, sight, hearing, and motor function at a level where they could complete the tasks, and no known history of autism spectrum disorder (ASD), brain damage, or epilepsy. Additionally, children referred to the ADHD clinic had to receive an (ICD‐10) ADHD diagnosis based on thorough standard clinical and multidisciplinary procedures at the hospital using several informants (parents, teachers, and the child), the Schedule for Affective Disorders and Schizophrenia for School‐aged Children, Present and Lifetime Version (Kaufman & Schweder, 2004), and when indicated, supplementary neuropsychological assessment and the Autism Diagnostic Observation Schedule (Lord et al., 1989). Additionally, all children were assessed with the ADHD Rating Scale (DuPaul et al., 1998). For children with ADHD taking stimulant medication (*n* = 22), it had to be discontinued ≥ 24 h before assessment. The NCs could not have a previous or current ADHD diagnosis based on parent report. A total of 10 children in the possible ADHD group were excluded due to ASD, premature birth, or IQ < 70. The final sample consisted of 46 children with ADHD (37 boys [80.4%], mean age 9.69 [SD = 1.17]) and 40 NCs (17 boys [42.5%], mean age 9.22 [SD = 1.41]). According to parent reports, none of the included children had a language disorder diagnosis. A flow chart illustrating the inclusion process is included in Supporting Information S1: Figure S1.

### Procedures

The data were collected between May 2018 and December 2021. Legal guardians of children with possible ADHD received invitation letters via mail, while legal guardians of NCs were invited via school intranets, social media campaigns, or webpages relevant to parents of school‐age children. Diagnoses in the ADHD group were based on standard clinical and multidisciplinary assessment at the specialist ADHD clinic. Cognitive assessment was carried out in two sessions (1.5 h each, approx. 2 weeks apart) in a quiet room by a trained examiner and the children received a small gift (value approx. 7 euro) at the end of the final session. Parents completed an extensive questionnaire battery. Here we report on ADHD behavior and background information. If parents consented, questionnaires were also sent to the child's primary teacher. Due to low teacher response rates during the Covid‐19 pandemic and resulting lockdown, available teacher data were included to assess questionnaire interrater agreement between parents and teachers.

### Measures, Administration, and Coding

Language measures included in the current study were chosen because they are considered gold standard measures of narrative language production (Jepsen et al., 2022) and core language (Korrel et al., 2017; Tannock, 2018). Executive function measures were chosen as they have previously been found to differentiate between children with ADHD and neurotypical children (see e.g., Cortese et al., 2015; Kofler et al., 2013; Ramos et al., 2020; Snyder et al., 2015; Pievsky & McGrath, 2018).

A detailed description of all measures and the narrative coding scheme is included in Supporting Information S1: Table S1–S5. Narrative language: The children's stories were elicited with the wordless picture book “Frog, where are you?” by Mayer (1969), which is widely used in narrative language research (Flory et al., 2006; Norbury & Bishop, 2003). The book is about a boy on the search for his pet frog that has run away. The child was informed that the examiner did not know the book, and before being encouraged to tell the story, the child was given a few minutes to become familiar with the book, while the examiner looked away. This procedure is considered to capture narrative production in real time, independently of memory or scaffolding from a previously heard story (Flory et al., 2006; Stirling et al., 2014). The stories were audiotaped for later transcription and coding. Two independent raters blind to child diagnostic status transcribed the children's narratives verbatim (following Brynskov et al., n.d.) and coded the narratives based on an a priori created scheme adapted from and supported in Jepsen et al. (2022) focusing on nine narrative outcomes (covering five aspects; see below). The independent raters were trained in the transcription and coding manuals by the first author, ongoing reliability checks of transcription and coding were performed, and raters received ongoing supervision from the first, second, and last author. At the end of the coding process, a final reliability check between the two raters was performed with a two‐way random ICC analysis (ICC(2), k) using randomly chosen transcripts (35%), and was excellent for all nine outcomes (>0.90).

*Coherence* refers to description of the story’s beginning, middle, and end, each of which was rated on a scale of 0–2 with higher scores indicating a more coherent story (adapted from Norbury & Bishop, 2003). Outcome was the summed coherence score (max. 6 points).

*Cohesion* includes *ambiguous references* (i.e., it is unclear which character or place the child is referring to; Liles et al., 1989) and *causal conjunctions* (e.g., because, then; Kuijper et al., 2017). Outcomes were total number of ambiguous references and total number of causal conjunctions.

*Disruptive errors* are instances when a respondent tells an event *out of sequence*, makes an *irrelevant comment* (i.e., unrelated to the story), or *misinterprets story events* (Tannock et al., 1993). Outcomes were total number of events told out of sequence, total number of irrelevant comments, and total number of misinterpreted story events.

*Syntactic complexity* includes *morpho‐syntactic errors* (e.g., incorrect inflection of a word or clause; Baixauli Fortea et al., 2018) and *mean length of utterances* (MLU) calculated by dividing the total number of words in the narrative with the total number of communication‐units, and greater MLU indicating greater language complexity (Potratz et al., 2022). Outcomes were total number of morpho‐syntactic errors and MLU.

*Internal state language* (ISL) is any *reference to characters' internal states* (e.g., happy, thinking, believing), and outcome was total number of internal state references (Siller et al., 2014).

Executive function: Three computer‐tasks were administered to measure WMU; a 2‐back spatial task (adapted from Friedman et al., 2008), Mental Counters (Huizinga et al., 2006), and the Tic Tac Toe task (Huizinga et al., 2006). The outcome from each task was mean accuracy. Three computer‐tasks were also administered to measure IN; the Stop‐Signal task (Logan, 1994; Logan et al., 1997; Williams et al., 1999), the Flanker task (Huyser et al., 2011), and the Go No Go task (GNG; Tsujimoto et al., 2007). The outcomes were stop signal reaction time (SSRT) in ms from the Stop‐Signal task (Huizinga et al., 2006; Verbruggen et al., 2019), the median RT in ms on incongruent trials from the Flanker task (Huizinga et al., 2006), and percent false alarms from the GNG (Young et al., 2018).

Core language: Four tasks (Concepts and Following Directions, Formulated Sentences, Word structure [<age 9], and Word Classes 2 [>age 9]) from the Core Language Index from the Clinical Evaluation of Language Fundamentals‐4 (CELF‐4; Semel et al., 2003) were administered to measure core language. Outcome was the core language summary score (i.e., the overall sum of the age‐normed scaled scores). The Scandinavian versions of these CELF‐4 subtests have been found to have acceptable to good internal consistency (Semel et al., 2003).

General cognitive function: The Raven's Colored Progressive Matrices (CPM) (Raven's CPM), a nonverbal pattern recognition task, was administered to measure general cognitive function. Previously, the standardized score from the Raven's CPM has been found to have good internal consistency and construct validity (Cotton et al., 2005; Raven, 2000).

ADHD behavior: The 18‐item parent version of the Strengths and Weaknesses of Attention Deficit/Hyperactivity Disorders and Normal Behavior (SWAN; Swanson et al., 2012) was administered to measure perceived ADHD behavior in the child. Outcome was the mean of all 18 items with higher scores indicating more perceived ADHD behavior. In the present study, and in line with previous studies (e.g., Arnett et al., 2013), the Strengths and Weaknesses Assessment of Attention Deficit/Hyperactivity Disorders and Normal Behavior was found to have good to excellent internal consistency (McDonald's omega_ADHD_ = 0.88, omega_NC_ = 0.95) and good interrater reliability between parents and teachers (ADHD, *r* = 0.57, *n* = 25, *p =* 0.003; neurotypical comparison children (NC), *r* = 0.61, *n* = 20, *p* = 0.004).

Background information: Information about the child's age and medical and mental health history as well as parental education (defined as length of education in years [range 0–17.5] from the parent with the longest education) was collected through a questionnaire.

### Data preparation

First, task and questionnaire data were inspected for missing data points and outliers. For task data, missing at case‐level was <5% (e.g., due to refusal to participate or computer malfunction) and 2.5%–12.6% at item‐level for individual tasks. For questionnaire data, missing at case‐level was 2.3% and 0.5% at item‐level. Little's MCAR test indicated that all missing data points were missing completely at random. Visual inspection flagged no extreme outliers. Second, one narrative outcome, “Out of sequence,” was excluded due to lack of data (i.e., very few [*n* = 9] children made this error). All narrative outcomes were included individually in the analyses (as outcomes considered to measure the same narrative language aspect did not correlate significantly; see Supporting Information S1: Table S6). Third, executive function data were cleaned, resulting in all trials with a RT less than 120 ms being removed as they indicate responses too fast for appropriate processing of a stimulus to have occurred (Berger & Kiefer, 2021). Additionally, as the Flanker task is an RT task, all trials with a *z*‐score more than 3 ± the child's individual mean RT (on correct incongruent trials) were excluded (<1%; Huizinga et al., 2006). In the stop‐signal task (and following Verbruggen et al., 2019), SSRT was only calculated if mean RT on go trials was longer than mean RT on unsuccessful stop trials, and if stop percentage was 25%–75% (<9% excluded). Finally, in line with current recommendations (see e.g., Snyder et al., 2015), significantly correlated outcomes from EF measures were combined. As the GNG did not correlate significantly with the remaining IN tasks (see Supporting Information S1: Table S7), it was excluded from further analysis. The remaining scores from the WMU and the IN tasks were transformed to z‐scores based on the NC group's mean and SD, and combined to form one WMU and one IN outcome.

### Data analysis

The analysis was performed in two linked phases. In the first phase, the associations between ADHD and the different narrative outcomes were examined using a model that specified all eight narrative outcomes as the dependent variables being regressed on variables representing diagnostic status (ADHD/NC), age, and sex as the independent variables. Significant regression coefficients indicate the mean differences between the ADHD and NC groups controlling for age and sex. In the second phase, if a statistically significant association between diagnostic status and any narrative outcome was found (adjusting for age and sex), bootstrapped multiple mediation analysis was performed with diagnostic status as the independent variable and the narrative outcome as the dependent variable, and WMU, IN, and core language as mediators. Age and sex were also included in the mediation model to control for possible age and sex effects on the mediators as well as the predictor (Muthen et al., 2016). General cognitive functioning was not controlled for as this may eliminate variance that is a result of ADHD (Mackenzie & Wonders, 2016) and produce overcorrected results (Dennis et al., 2009). An additional analysis including social economic status (SES; measured as parental education in years) as a covariate were conducted as well. The residual variances for the mediators were allowed to correlate in the models. The total indirect and the specific indirect effects of the proposed mediators as well as the bootstrapped 95% confidence intervals (CI) were examined. In all analyses, the significance levels (*p*‐values) were reported based on the unstandardized regression coefficients (*B*‐values), but standardized values (*β*) were also reported. Given the binary nature of the diagnostic status and sex variables, the standardized regression coefficients were reported as “y‐standardised,” where the latent variable (*y*) is standardised, but the predictor variables (diagnostic status, sex) retain their binary coding (Muthen et al., 2016). Hence, the effect can be interpreted as the mean difference between the two levels of the binary variable in terms of a standard deviation of a standard normal distribution. Analyses were performed using Mplus 8.8 (Muthen et al., 2016).

## RESULTS

Sample characteristics are presented in Table 1 below, and descriptives of tasks in Supporting Information S1: Table S8. Children in the NC group were significantly more likely to be female, and had lower levels of parent‐reported ADHD behavior, a higher mean IQ, as well as parents with a longer mean education than children in the ADHD group.

**TABLE 1:** Sample characteristics.

Characteristics	ADHD (*n* = 46)	NC (*n* = 40)	*t* or *x* ^*2*^	*p*	Effect size^a^
Age (*Mean* [SD])	9.69 (1.17)	9.22 (1.41)	−1.69	0.10	0.36
Sex (*n*)			13.18	<0.001	0.39
Boys	37	17			
Girls	9	23			
SWAN total score (*Mean* [SD])^b^	1.40 (0.62)	−0.76 (0.82)	−13.36	<0.001	−2.98
Raven's CPM standard score (*Mean* [SD])^c^	100.68 (16.83)	111.00 (15.82)	2.89	0.005	0.63
Co‐occurring ICD‐10 diagnoses (*n*)					
F81‐89 (excluding F84)	18	‐	‐	‐	‐
F95x Other behavioral and/or emotional disorder	≤5	‐	‐	‐	‐
F85 tic disorder	≤5	‐	‐	‐	‐
Parental education in years (*Mean* [SD])^d^	13.94 (2.34)	15.74 (2.27)	−3.47	<0.001	−0.78

Abbreviations: CPM, Colored Progressive Matrices; ICD‐10, Internal Classification of Diseases‐10; NC, neurotypical comparison children; SD, standard deviation, SWAN, Strengths and Weaknesses Assessment of Attention Deficit/Hyperactivity Disorders and Normal Behavior.

^a^ Cohen's *d* or Phi. Cohen's *d* small effect = 0.20, medium effect = 0.50, large effect ≥0.80 (Cohen, 1988); Phi small effect = 0.1, medium effect = 0.3, large effect ≥ 0.5 (Field, 2007).

^b^ ADHD (*n* = 45); NC (*n* = 39).

^c^ ADHD (*n* = 44); NC (*n* = 40).

^d^ ADHD (*n* = 43); NC (*n* = 37).

### The association between ADHD and narrative language production

The results from the multivariate multiple regression analysis are presented in Table 2. The overall prediction model was statistically significant *χ* ^*2*^ (24) = 67.85, *p* < 0.01, indicating that diagnostic status, age, and sex explained a significant amount of the collective variance in narrative production. Diagnostic status, adjusting for age and sex, explained a significant proportion of the variance in three of eight narrative outcomes, with children with ADHD producing significantly more ambiguous references (*β* = 0.72, *p* < 0.001), less coherent narratives (*β* = −0.59, *p* = 0.001), and more morpho‐syntactic errors (*β* = 0.47, *p* = 0.015) compared to NCs.

**TABLE 2:** Multivariate multiple regression model with eight narrative language outcomes as the dependent variables and diagnostic status, age, and sex as the independent variables.

Narrative Aspect	Dependent variable	Independent variable	Unstand.				Stand.	
			B	*S.E.*	*z*	*p*	*β* ^a^	*R* ^*2*^
Coherence	Coherence	Diagnostic status	−0.712	0.222	−3.200	0.001	−0.586	0.217***
		Age	0.280	0.091	3.078	0.002	0.231	
		Sex	0.589	0.242	2.433	0.015	0.485	
Cohesion	Ambiguous references	Diagnostic status	1.699	0.413	4.118	<0.001	0.724	0.158**
		Age	−0.431	0.210	−2.055	0.040	0.184	
		Sex	−0.062	0.438	−0.142	0.887	−0.027	
	Causal conjunctions	Diagnostic status	−0.235	2.411	−0.098	0.922	−0.021	0.008
		Age	−0.769	0.988	−0.778	0.436	−0.068	
		Sex	0.034	2.515	0.013	0.989	0.003	
Disruptions	Irrelevant comments	Diagnostic status	−0.849	1.063	−0.798	0.425	−0.204	0.038
		Age	0.584	0.381	1.534	0.125	0.141	
		Sex	−0.267	0.982	−0.272	0.786	−0.064	
	Misattributions	Diagnostic status	0.852	0.443	1.925	0.054	0.380	0.031
		Age	−0.022	0.181	−0.119	0.905	−0.010	
		Sex	0.252	0.448	0.562	0.574	0.112	
Syntactic complexity	Morpho‐syntactic errors	Diagnostic status	1.359	0.621	2.189	0.029	0.468	0.124*
		Age	−0.665	0.266	−2.498	0.012	−0.229	
		Sex	−0.380	0.659	−0.577	0.564	−0.131	
	MLU	Diagnostic status	−0.407	0.328	−1.242	0.214	−0.273	0.246**
		Age	0.542	0.109	4.971	<0.001	0.363	
		Sex	0.753	0.332	2.268	0.023	0.504	
ISL	ISL	Diagnostic status Age Sex	−0.566	0.816	−0.694	0.488	−0.142	0.056
			0.653	0.347	1.880	0.060	0.163	
			1.101	1.001	1.099	0.272	0.275	

Abbreviations: ISL, internal state language; MLU, mean length of utterance.

^a^ y‐standardization.

*p < 0.05; **p < 0.01; ***p = 0.001.

Age explained a significant amount of the variance in four of eight narrative outcomes, suggesting that older children have longer MLUs (*β* = 0.36, *p* < 0.001), and produce more coherent narratives (*β* = 0.23, *p* < 0.001), fewer morpho‐syntactic errors (*β* = −0.23, *p* = 0.001), and fewer ambiguous references (*β* = −0.18, *p* = 0.05) than younger children. Sex explained a significant amount of the variance in two of eight narrative outcomes, suggesting that girls have longer MLUs (*β* = 0.50, *p* = 0.02) and produce more coherent narratives (*β* = 0.49, *p* < 0.05) than boys. Diagnostic status, age, and sex did not significantly explain any of the variance in the remaining categories (i.e., causal conjunctions, irrelevant comments, misinterpreting events, or ISL).

### Multiple mediation of the association between ADHD and narrative language production

As children with ADHD were found to produce narratives that contained significantly more ambiguous references, were less coherent, and contained more morpho‐syntactic errors than NCs in the regression analysis, a bootstrapped multiple mediation analysis was conducted for each of these narrative outcomes. The direct effect of diagnostic status (adjusting for age and sex) on the mediators were: WMU *β* = −0.842, *p* < 0.001, IN *β =* 0.192, *p* = 0.45, and core language *β = −*0.90, *p* < 0.001, indicating that the children in the ADHD group obtained lower scores on WMU and core language (but not IN) tasks than NCs.

**FIGURE 1:**
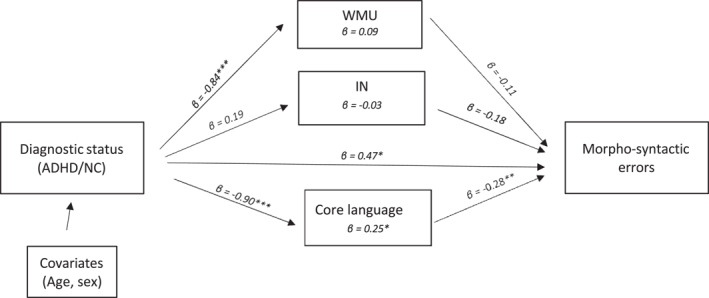
Multiple mediation model of the effect of working memory, inhibition, and core language on the association between ADHD and morpho‐syntactic errors. IN = response inhibition; NC = Neurotypical comparison children; WMU = Working Memory Updating. *p < 0.05; **p < 0.01; ***p < 0.001.

For the association between diagnostic status and morpho‐syntactic errors (adjusting for age and sex), the total indirect effect was not statistically significant, but core language had a significant specific indirect effect on the production of morpho‐syntatic errors (*β* = 0.25, *p* = 0.03, 95% CI = [0.075, 0.509]; see Table 3 and Figure 1). This indicates that core language partially mediates the association between diagnostic status and the production of morpho‐syntactic errors. No other indirect effects were statistically significant.

**TABLE 3:** Multiple mediation model of the effect of WMU, IN and core language on the association between ADHD and morpho‐syntactic errors.

			Unstand.			Stand.	
	*B*	*SE*	*z*	*CI* ^a^	*p*	*β* ^b^	*CI β* ^a^
Effects of diagnostic status on morpho‐syntactic errors (controlling for age and sex)							
Total effect	1.372	0.632	2.170	[0.239, 2.800]	0.030	0.472	[0.071, 0.851]
Total indirect	0.904	0.514	1.759	[1.101, 2.148]	0.079	0.311	[0.031, 0.616]
Total direct	0.467	0.632	0.739	[−0.805, 1.738]	0.460	0.161	[−0.272, 0.621]
Specific indirect effects							
Mediator							
WMU	0.257	0.375	0.686	[−0.301, 1.258]	0.493	0.088	[−0.119, 0.374]
IN	−0.093	0.175	−0.530	[−0.584, 0.182]	0.596	−0.032	[−0.219, 0.063]
Core language	0.740	0.342	2.164	[0.222, 1.619]	0.030	0.254	[0.075, 0.509]

Abbreviations: CI, bootstrapped confidence intervals; IN, response inhibition; SE, standard error; WMU, working memory updating.

^a^ 95% CIs.

^b^ y‐standardization.

Neither the total nor the specific indirect effects on coherence (see Table 4 and Supporting Information S1: Figure S2) or ambiguous references (see Table 5 and Supporting Information S1: Figure S3) were statistically significant, which was supported by inspection of the 95% CIs, where all indirect effects contained zero. Consequently, WMU, IN, and core language do not appear to mediate the association between diagnostic status and these narrative outcomes.

**TABLE 4:** Multiple mediation model of the effect of WMU, IN and core language on the association between ADHD and narrative coherence.

		Unstand.				Stand.	
	*B*	*SE*	*z*	*CI* ^a^	*p*	*β* ^b^	*CI β* ^a^
Effects of diagnostic status on coherence (controlling for age and sex)							
Total effect	−0.713	0.226	−3.161	[−1.139, −0.251]	0.002	−0.587	[−0.886, −0.205]
Total indirect	−0.122	0.170	−0.719	[−0.507, 0.171]	0.472	−0.101	[−0.405, 0.147]
Total direct	−0.591	0.286	−2.063	[−1.109, 0.029]	0.039	−0.486	[−0.904, 0.028]
Specific indirect effects							
Mediator							
WMU	−0.010	0.160	−0.060	[−0.316, 0.315]	0.952	−0.008	[−0.265, 0.255]
IN	−0.024	0.062	−0.395	[−0.213, 0.052]	0.693	−0.020	[−0.171, 0.046]
Core language	−0.088	0.176	−0.503	[−0.501, 0.213]	0.615	−0.073	[−0.407, 0.190]

Abbreviations: CI, bootstrapped confidence intervals; IN, response inhibition; SE, standard error; WMU, working memory updating.

^a^ 95% CIs.

^b^ *y*‐standardization.

**TABLE 5:** Multiple mediation model of the effect of WMU, IN and core language on the association between ADHD and ambiguous references.

		Unstand.				Stand.	
	*B*	*SE*	*z*	*CI* ^a^	*p*	*β* ^b^	*CI β* ^a^
Effects of diagnostic status on ambiguous references (controlling for age and sex)							
Total effect	1.707	0.420	4.064	[0.967, 2.573]	<0.001	0.727	[0.387, 1.040]
Total indirect	0.467	0.341	1.368	[−1.125, 1.211]	0.171	0.199	[−0.060, 0.495]
Total direct	1.240	0.491	2.525	[0.296, 2.188]	0.012	0.528	[0.085, 0.935]
Specific indirect effects							
Mediator							
WMU	0.077	0.286	0.270	[−0.494, 0.632]	0.787	0.033	[−0.245, 0.263]
IN	−0.095	0.150	−0.636	[−0.540, 0.105]	0.525	−0.041	[−0.215, 0.051]
Core language	0.485	0.292	1.661	[−0.016, 1.170]	0.097	0.206	[−0.010, 0.484]

Abbreviations: CI, bootstrapped confidence intervals; IN, response inhibition; SE, standard error; WMU, working memory updating.

^a^ 95% CIs.

^b^ y‐standardization.

Sensitivity analyses excluding one sibling in each sibling pair (*n* = 4) in the NC group did not significantly change the results (not reported). Additional analyses including SES as a covariate yielded the same overall results (see Supporting Information S1: Tables S9–S12).

## DISCUSSION

The present study examined the association between ADHD and narrative language production, and whether it was mediated by executive function and/or core language. While associations between ADHD and several aspects of narrative language production were found, executive function did not mediate any of them. Core language partly mediated the association between ADHD and morpho‐syntactic errors, suggesting that core language difficulties contribute to the production of grammatical errors when telling a story. Given the exploratory nature of the study, the results are in need of replication.

Consistent with our hypothesis and previous findings (see Jepsen et al., 2022), children with ADHD produced more ambiguous references, less coherent narratives, and more morpho‐syntactic errors than NCs. When telling a story, the use of appropriate referencing (e.g., correct pronouns), inclusion of the central parts of a story (e.g., the start, middle, and end), and the use of correct morpho‐syntax (e.g., correct inflection of words), are important for the story to be understandable to the listener (Liles et al., 1989; Norbury & Bishop, 2003). Consequently, difficulties with these aspects of narrative language could mean that everyday speech is difficult to follow. Furthermore, appropriate referencing and correct morpho‐syntax are both fundamental grammatical skills (Kuijper et al., 2017; Liles et al., 1995; Parigger, 2012), suggesting children with ADHD may have difficulty with grammar. This would be in line with a previous meta‐analysis, where children with ADHD were found to obtain lower scores than NCs on standardized tasks covering grammar and structural aspects of language (Korrel et al., 2017). Finally, increased morpho‐syntactic error production has been found in children with developmental language disorders (Christensen, 2019), and it is possible that the presence of these error types in the ADHD group are indicative of more general language difficulties. Overall, the results from the current study suggest that children with ADHD experience difficulties with several central aspects of narrative language production that may result in impaired everyday speech and require targeted language support.

In contrast to our hypothesis, ADHD was not associated with causal conjunctions, misattributions, irrelevant comments, ISL, or MLU. This was somewhat surprising as a previous meta‐analysis (Jepsen et al., 2022) supported an association between ADHD and these aspects of narrative language. However, very few studies have examined these narrative outcomes to date (*N* range = 1–5; Baixauli Fortea et al., 2018; Bergman & Hallin, 2021; Koltun, 2004; Kuijper et al., 2017; Parigger, 2012; Purvis & Tannock, 1997; Rumpf et al., 2012; Tannock et al., 1993). Further, inspection of these studies suggests that in the studies where the groups where matched on age, sex, as well as other relevant factors such as general language abilities, children with ADHD and NC did not differ significantly with respect to these narrative outcomes. However, these outcomes may also be affected by how the stories were elicited, for example, some error types such as misattributions or irrelevant comments may be more likely to occur in recall tasks than online narrative tasks where the story is constructed by the child in real time. While more research on the association between ADHD and causal conjunctions, misattributions, irrelevant comments, ISL, and MLU is clearly needed (and where relevant factors such as age and sex are taken into account), the results could suggest that children with ADHD may be comparable to children without ADHD with respect to many narrative aspects.

Neither WMU nor IN mediated the association between ADHD and narrative language production (i.e., coherence, ambiguous references, and morpho‐syntactic errors) in the present study. This contrasts the dominating hypothesis in the field stating that executive function deficits are the primary cause of narrative language difficulties in children with ADHD (Tannock & Schachar, 1996; Westby & Watson, 2021). The findings are in line with three previous studies (Flory et al., 2006; Koltun, 2004; Parigger, 2012), but contradict two previous studies (based on the same sample; Kuijper et al., 2017; Kuijper et al., 2015). In sum, four (including the current study) of the 6 studies that have examined the role of executive function in relation the narrative production in ADHD to date, have not found executive function to explain narrative language difficulties in children with ADHD. Consequently, the current evidence does not support executive function as the main contributor to the narrative language production of children with ADHD.

This poses the question of what, if not executive function, potentially mediates the association between ADHD and narrative language production? We found core language to partially mediate the association between ADHD and morpho‐syntactic errors, suggesting that core language difficulties may contribute to increased production of morpho‐syntactic errors in the ADHD group. This is in line with findings from two previous studies (Baixauli Fortea et al., 2018; Rumpf et al., 2012). However, core language did not mediate the association between ADHD and coherence and ambiguous references, respectively. While speculative (due to the lack of research), it is possible that as we fail to find support for the contribution of underlying cognitive skills to the narrative language production of children with ADHD, we may need to examine contextual factors. For instance, the narrative language production of children with ADHD may have more to do with the availability of opportunities for listening to and practicing telling stories during development (e.g., at home and in school) than any key cognitive process in isolation. However, it is also possible that other cognitive functions than WMU and IN have a role to play. For instance, difficulties in temporal processing may lead to a child telling a story out of order due to difficulties perceiving or representing time. Finally, investigating the impact of inattention and/or hyperactivity/impulsivity on the development of narrative language may also inform our understanding of the association between ADHD and narrative language. To determine how to best support children with ADHD in their narrative language development, future studies should examine an array of factors that may influence this development, including cognitive functions, ADHD behavior, as well as home literacy activities or time taken to share personal experiences in school and at home.

This study had several strengths, including being one of the largest studies to examine the association between ADHD, executive function, and narrative language to date, the use of a previously supported categorization system to code narrative language abilities, blinded coding of narratives, inclusion of several measures of each executive function domain, and adjustment for age and sex in the analyses. There are also several limitations. First, due to the cross‐sectional design of the study, the causal relation between ADHD, executive functions, core language, and narrative production remains speculative, and needs to be examined in longitudinal studies. Future longitudinal studies may additionally benefit from examining this relationship at different stages of development. Second, while large for this type of study, the sample size limits the robustness of the results, and they require replication. Third, the current study investigated ADHD as a binary predictor. Future research should consider examining the association between narrative language production and ADHD using a dimensional model, and whether inattention and hyperactivity/impulsivity are differentially associated with narrative language. This could provide potentially important information for future efforts to tailor support for children with ADHD and co‐occurring narrative language difficulties. Fourth, different results may have emerged if a different narrative task or different executive function tasks had been included in the study (e.g., tasks relying more heavily on verbal working memory). Replication, investigating narrative language production of children with ADHD during different narrative tasks, may also be important in future research. Fifth, though none of the children in the current ADHD group had a co‐occurring language disorder, considering the high rate of co‐occurring language disorders in children with ADHD (see e.g., Sciberras et al., 2014) the role of co‐occurring language disorder should be examined in future studies. Finally, the Covid‐19 lock‐down happened during data collection, which likely made several participants opt out of participating, however, we have no available data to compare responders and non‐responders. This also resulted in very few teachers returning questionnaires (e.g., only 26% of teachers of children with ADHD completed the Development and Well Being Assessment [DAWBA; Goodman et al., 2000]), and we had to rely on hospital diagnosis and parent reports. However, agreement between available DAWBAs and hospital ADHD diagnoses was 87% (ADHD group), and in the NC group no DAWBA indicated an ADHD diagnosis, corresponding with parent reports.

## CONCLUSION

The current study found that in children aged 7–11, ADHD was associated with producing narrative language that was less coherent and contained more ambiguous references and grammatical errors. While executive function did not mediate this association, core language partly mediated the association between ADHD and the production of morpho‐syntactic errors. This suggests that narrative language difficulties in children with ADHD are not manifestations of executive function deficits. Consequently, clinicians may need to be aware of language during assessment and support for children with ADHD. Support for children with ADHD and co‐occurring narrative language difficulties may need to target core language to improve structural aspects of the story such as morphosyntactic errors. Although many factors, including executive function, should be considered when examining and supporting children with ADHD, additional research is needed to identify the specific factors affecting the challenges children with ADHD have producing coherent stories.

## AUTHOR CONTRIBUTIONS

**Ida Bonnerup Jepsen**: Conceptualization; data curation; formal analysis; funding acquisition; investigation; methodology; project administration; software; visualization; writing—original draft; writing—review and editing. **Cecilia Brynskov**: Conceptualization; methodology; supervision; writing—review and editing. **Per Hove Thomsen**:Data curation; methodology; writing—review and editing. **Charlotte Ulrikka Rask**: Data curation, writing—review and editing. **Rikke Lambek**: Conceptualization; formal analysis; investigation; methodology; supervision; writing—review and editing.

## CONFLICT OF INTEREST STATEMENT

I.B.J. (first author) and R.L. (fifth author) have received funding from TrygFonden and Fru C. Hermansens foundation. P.H.T. (third author) has received grants from TAKEDA and speaker's fee from Medice within the last 5 years. He receives royalties from Danish publishers from books on child and adolescent psychiatry.

## ETHICAL CONSIDERATIONS

The study was approved by the Danish regional ethics committee (1‐10‐72‐443‐17) and registered with the Danish data protection agency (2016−051−000001) and clinical trials (NCT03917316). All legal guardians received written and verbal information about the study and gave written and verbal consent, while children gave verbal assent.

## Supporting information

Supporting Information S1

## Data Availability

Research data are not shared due to Danish law (GDPR).
