# Supplementary material for: The association between attention‐deficit/hyperactivity disorder and narrative language: What is the role of executive function?
Source: JCPP Adv. 2025 Feb 25;5(4):e70007. doi: 10.1002/jcv2.70007 (PMC12698281; doi:10.1002/jcv2.70007)
Supplement: Supplementary file 1 — Supporting Information S1 [file JCV2-5-e70007-s001.docx]

**Journal of Child Psychology and Psychiatry**

Online Supporting Information

The Association Between Attention-Deficit/Hyperactivity Disorder and Narrative Language:

What is the Role of Executive Function?

Ida Bonnerup Jepsen^1^, Cecilia Brynskov^2^, Per Hove Thomsen^3^, Charlotte Ulrikka Rask^3^, and Rikke Lambek^1^.

^1^Department of Psychology and Behavioural Sciences, Aarhus University, Denmark

^2^Department of Nordic Studies and Linguistics, University of Copenhagen, Denmark

^3^Research unit, Department of Child and Adolescent Psychiatry, Aarhus University Hospital Psychiatry, and Department of Clinical Medicine, Aarhus University, Denmark

Neurotypical comparison children (NC)

ADHD

Invited to participation

(*n* = n/a)

Invited to participation

(*n* = 746)

Agreed to a phone call with verbal information (*n* = 77)

Agreed to a phone call with verbal information (*n* = 98)

Agreed to first meeting (*n* = 46)

Agreed to first meeting (*n* = 62)

Dropped out (*n* = 6)

Dropped out (*n* = 6)

Participated (*n* = 56)

Excluded due to ASD, premature birth, or IQ<70

(*n* = 10)

NC group (*n* = 40)

ADHD group (*n* = 46)

**Figure S1**. Sample flow chart

*Note*. ASD = autism spectrum disorder; n/a = not available.

# Measures

# **Table S1**. Narrative task coding scheme

| Narrative  aspect | Description | Coding | Outcome |
| --- | --- | --- | --- |
| Coherence. | Refers to the child’s global presentation of the story’s meaning as well as temporal and causal overarching structure (Baixauli et al., 2016; Norbury & Bishop, 2003). Operationalized as adequate description of the story’s beginning, middle, and end (Norbury & Bishop, 2003). | *Beginning*: 2 points awarded if the story included that the frog ran away, and the boy looked for the frog in his room, 1 point if only one of these aspects were included in the story, and 0 if none of them were included.  *Middle*: 2 points awarded if the story included that the boy engaged in at least three separate attempts to find the frog, 1 point if 1-2 attempts were made, and 0 points if no attempts were made.  *Ending*: 2 points awarded if the story included that the boy finds his OWN frog, *and* that he takes a (baby)frog home, 1 point if only one of these aspects was included in the story, and 0 if none of them were included (adapted from Norbury & Bishop, 2003). | Total coherence score (max 6 points). |
| Cohesion. | Cohesive devices are tools to attain coherence in that they link sentences together (Norbury & Bishop, 2003). Operationalized as ambiguous references (Liles & Purcell, 1987) and causal conjunctions (Kuijper et al., 2017). | An *ambiguous reference* is coded when it is ambiguous what character, event, or place is being referred to either due to (i) the character not having been properly introduced (e.g., saying “he” without having introduced “the boy”), (ii) pronouns are used ambiguously (e.g., saying “it goes away” when there are two or more characters that could be “it”), or (iii) wrong referent (e.g., “her” instead of “he”).  *Causal conjunctions* were coded (e.g., because, therefore, and then, etc.). | Total number of ambiguous references in the story.  Total number of causal conjunctions in the story. |
| Disruptions. | Disruptions encompass mistakes or errors in the story that disturb the overall coherence (Flory et al., 2006; Tannock et al., 1993) and are operationalized as instances where the child tells events out of sequence, provide irrelevant comments, and misinterprets story events (Tannock et al., 1993). | *Out of sequence (OOS)* were coded when a child told events in the wrong order or talked about something that happened in a previous but not current picture.  *Irrelevant comments* were coded when the child stepped out of the storytelling-role to comment on his/her own life/knowledge or to evaluate how well he/she was performing the task etc.  *Misattributions* were coded when the child misinterpreted something that took place in the story (e.g., that the dog jumped out the window, when it actually fell; Tannock et al., 1993). | Total number of OOS errors in the story.  Total number of irrelevant comments in the story.  Total number of misattributions in the story. |
| Syntactical complexity. | Syntactical complexity refers to the level of complexity of the sentences the child produces as well as how many morpho-syntactic errors the child produces. Operationalized as mean length utterance (MLU) and morpho-syntactic errors (Baixauli Fortea et al., 2018; Potratz et al., 2022). | *MLU* was calculated from total number of words divided by total number of communication-units (Potratz et al., 2022). A communication-unit (C-unit) was defined as a sentence with subject-verb-object, or as exclamations.  *A morpho-syntactic error* was coded when the child produced a wrong inflection of a word or a clause (e.g., “eated” instead of “ate”), or if a sentence was lacking a clause in order to be correct (Baixauli Fortea et al., 2018). | MLU.  Total number of morpho-syntactic errors in the story. |
| Internal state language (ISL). | This category refers to talk about perceptions, thoughts, beliefs, and feelings, that is, states that cannot be observed directly but have to be inferred from the characters actions and facial expression (Rumpf et al., 2012). Operationalized as references to internal states (Rumpf et al., 2012). | *ISL* was coded every time the child used a term that referred to characters’ internal states such as angry, sad, happy, think, know etc. (Siller et al., 2014). | Total number of ISL referents. |

*Note*. ISL = Internal State Language; MLU = Mean Length Utterance; OOS = Out of Sequence.

# **Table S2**. Executive function measures

| Domain | Procedure | Nr. of trials | Interstimulus and response times | Outcome |
| --- | --- | --- | --- | --- |
| Working memory updating (WMU):  2-back spatial task (modelled from Friedman et al., 2008's Spatial 2-back task). | In this task there were 10 open squares scattered randomly across the screen. Then one box at a time became solid blue for 500 ms, giving the appearance that it flashed. For each flash the child had to press a button representing either “yes” indicating that the box that flashed was the same that flashed two trials earlier, or “no” indicating that it was not the same box (“yes” to “no” ratio was 1:3). There were no instances where the current flashing square was the same as the one that flashed one or three trials back. The task was piloted with neurotypical children and there was a large correlation (*r* = .79, n = 11, *p<*.001) between scores on the 2-back spatial task and the Tic Tac Toe task (see below). | 9 practice trials, 2 x 24 test trials. | ISI: 1500 ms.  Presentation: 500 ms.  No feedback.  Response time (max): 2000 ms. | Mean accuracy (proportion of correct response across all trials [yes *and* no]). |
| WMU:  Mental Counters (Huizinga et al., 2006). | The child had to keep track of the values of two (blocked) independent “counters”. The counters consisted of a horizontal line, above or below which squares appeared. When a square appeared above the line, “1” was added to the value of the counter, and when a square appeared below the line, “1” was subtracted from the line. The child had to press a button when the result of one of the counters was one *more* than a pre-specified value (e.g., if the pre-specified value was 2, the child had to press the button when one of the two counters reached the value 3). | 5 practice trials, 16 test trials. | ISI: 800-1200 ms.  Presentation:1000 ms.  Feedback: 400 ms (‘+’ for correct answer; ‘-‘ for incorrect answer; ‘x’ for too late answer).  Response time (max): 3500 ms. | Mean accuracy. |
| WMU:  Tic Tac Toe task (Huizinga et al., 2006). | The task consisted of a memorizing phase and a recognition phase. During the memorizing phase a pattern of ‘X’s and ‘O’s was presented within a 3x3 grid. Working memory load varied by patterns consisting of three versus four letters (i.e., low versus high memory load). The child initiated the recognition phase by pressing the space bar. During this phase ‘X’s and ‘O’s were presented one after another at different positions in the grid, in a series that varied from four to seven presentations for the low memory load and from four to nine presentations for the high memory load. The child had to press a button as soon as the pattern of ‘X’s and ‘O’s was the same as the pattern in the memorizing phase. | 5 practice trials, 2 x 15 trials. | ISI: 600-1000 ms.  Presentation: 600-1250 ms.  Feedback: 400 ms (‘+’ for correct answer; ‘-‘ for incorrect answer; ‘x’ for too late answer).  Response time (max): 3500 ms. | Mean accuracy. |
| Response inhibition: Go/No Go task (Tsujimoto et al., 2007). | The task started with a black screen, and then a blue or a green square, one at a time, appeared in the middle of the screen. Before the task begun, the child was encouraged to press the button as fast as possible as soon as the blue square appeared, however, to not press the button when the green square appeared. Following Young et al. (2018), the proportion of go to no go trials was 3:1, as this is the lowest proportion recommended in order to create false alarms [pressing the button when a green square appeared and thus not inhibiting the go response]. | 8 practice trials, 80 test trials. | ISI: 1000 ms.  Presentation: 100 ms.  No feedback.  Response time (max): 1000 ms. | False alarms in percent (commission errors; Snyder et al., 2015; Young et al., 2018). |
| Response inhibition:  Flanker task (Huyser et al., 2011). | The child was encouraged to focus on a central arrow to make a decision according to the direction of the central arrow while ignoring peripheral arrows. The task contained two conditions: congruent flankers, where all arrows pointed in the same direction [‘<<<<<’ or ‘>>>>>’], and incongruent flankers where the peripheral flankers pointed in a different direction than the central, target arrow [‘<<><<’ or ‘>><>>’]. | 50 practice trials, 100 test trials (50 congruent + 50 incongruent). | ISI: 1500 ms.  Presentation:  500 ms.  No feedback.  Response time (max): 1500 ms. | Median RT in ms for incongruent trials (Huizinga et al., 2006). |
| Response inhibition:  Stop-signal task (Logan, 1994; Logan et al., 1997; Williams et al., 1999). | In this task, ‘X’s and ‘O’s were presented in the center of the screen. Only one ‘X’ or one ‘O’ was presented at a time. When an ‘X’ appeared, the child had to press a button with an ‘X’ on it, and when an ‘O’ was presented on the screen, the child had to press a button with an ‘O’ on it). However, when a stop-signal sounded (a 100 ms 1000 Hz tone generated by the computer) the child had to withhold response by not pressing the button. | 32 practice trials, 5 x 32 test trials (24 go-signal trials and 8 stop signal trials). | ISI: 2500 ms.  Presentation: 1000 ms.  No feedback.  Response time: Stop signal delay started at 250 ms and was continually and automatically adjusted to the child (-50 ms each time the child correctly withheld response, and +50 ms each time the child did not. | Stop-signal reaction time (SSRT) in ms (mean reaction time to the go signal - mean stop signal delay). |

*Note*. ISI = Interstimulus Interval; SSRT = Stop Signal Reaction Time; WMU = Working Memory Updating.

# **Table S3**. CELF-4 language tests

| CELF-4 Task (Semel et al., 2003) | Language domain | Description | Outcome |
| --- | --- | --- | --- |
| Concepts and Following Directions . | Receptive language. | Each item contained a series of pictures and an instruction about which pictures to point to in a specific order. The items increased in difficulty. The task was discontinued if the child made four subsequent errors. The task contained 38 items (7-11 y.o.). | Total raw-score converted to age-normed scaled score. |
| Formulated Sentences. | Receptive and expressive language. | Each item consisted of a picture and a target word, and the child was encouraged to tell something about the picture using the target word. The child received between 0-2 points based on how grammatically and contextually correct the target word was used. The task was discontinued if the child produced four subsequent 0-point sentences. The task contained 22 items (7-11 y.o.) | Total raw-score converted to age-normed scaled score. |
| Word Classes (2). | Receptive and expressive language. | Each item contained a receptive and an expressive part. The child was presented with four words without visual support and was asked which two went together the best (receptive part), and *why* they went together the best (expressive part). The task was discontinued if the child made four subsequent errors on the receptive part. If the child’s answer to the receptive part was wrong, the child was presented with the correct answer and given the opportunity to answer the expressive part. The task contained 17 items (9-11 y.o.). | Total raw-scores (receptive + expressive part) converted to age-normed scaled scores and summed (receptive + expressive scaled score). |
| Word Structures. | Expressive and receptive language. | The task assessed the child’s knowledge of 10 (Danish) grammatical rules. For each new rule there was a picture where the examiner demonstrated the rule. The child was then encouraged to complete various sentences with visual prompts. It contained 39 items (7-8 y.o). | Total raw-score converted to age-normed scaled score. |

*Note.* CELF-4 = Clinical Evaluation of Language Fundamentals-4.

# **Table S4**. ADHD behavior

| Measure | Domain | Description | Responder | Outcome |
| --- | --- | --- | --- | --- |
| Strengths and Weaknesses of Attention-Deficit Hyperactivity Disorders symptoms and Normal behavior (SWAN; Swanson et al., 2012). | ADHD behavior. | SWAN is a parent and teacher-questionnaire that contains 18 items corresponding to the 18 symptoms (9 items for inattention and 9 items for hyperactivity/impulsivity) for ADHD listed in the Diagnostic and Statistical Manual of Mental Disorders-5 (APA, 2013). For each item, the respondent rates the child’s behavior on a 7-point Likert scale ranging from 3 (far below average) to -3 (far above average), such that higher scores indicate more perceived ADHD behavior (Swanson et al., 2012). | Parents and teachers. | Mean score on the 18 ADHD items. |

*Note*. SWAN = Strengths and Weaknesses of Attention-Deficit Hyperactivity Disorders symptoms and Normal behavior.

# **Table S5**. General cognitive function

| Measure | Domain | Description | Outcome |
| --- | --- | --- | --- |
| Raven’s colored progressive Matrices CPM (Raven, 2000). | General cognitive abilities. | Raven’s CPM is a non-verbal paper-pencil test that assesses general cognitive abilities. It is a pattern completion task increasing in difficulty. The child is encouraged to pick the correct solution out of six suggestions. The standardized score from the CPM correlated significantly with the full scale IQ from the WISC (Wechsler, 2003, 2014) obtained from hospital (*r* = .57, *n* = 38, *p*<0.01). The task contained 3 x 12 items. | Total raw score was converted to an age-normed standardized score. |

*Note*. CPM = Colored Progressive Matrices; WISC = Wechsler’s Intelligence Scale for Children.

# **Table S6**. Correlations between narrative outcomes

|  | Coherence | Ambiguous references | Causal conjunction | Irrelevant comments | Misa. | Mo-Sy Err. | MLU |
| --- | --- | --- | --- | --- | --- | --- | --- |
| 1. Coherence | - |  |  |  |  |  |  |
| 2. Ambiguous ref. | -.33** | - |  |  |  |  |  |
| 3. Causal conjunct. | .18 | -.16 | - |  |  |  |  |
| 4. Irrelevant comments | .01 | .15 | .24* | - |  |  |  |
| 5. Misattributions | -.29** | .30** | -.04 | .19 | - |  |  |
| 6. Mo-Sy. errors | -.13 | .51*** | .27* | .03 | .28** | - |  |
| 7. MLU | .36*** | -.14 | .37 | .02 | -.06 | -.16 | - |
| 8. ISL | .21 | .13 | .21 | .64*** | .18 | .12 | .10 |
| *Note*. Conjunct. = conjunctions, ISL = Internal state language, Misa = Misattributions, Mo-Sy Err = Morpho-Syntactic Errors, MLU = Mean Length Utterance, ref = references. | | | | | | | |
| **p*<.05 level, ***p*<.01 level, ****p*<.001 level. | | | | | | | |

# **Table S7**. Correlations between all executive function tasks

|  | Mental counters | Tic tac toe | 2-back | SSRT | Median RT incong. |
| --- | --- | --- | --- | --- | --- |
| 1. Mental Counters Mean acc. | - |  |  |  |  |
| 2. Tic Tac Toe Mean acc. | .58*** | - |  |  |  |
| 3. 2-back spatial task Mean acc. | .57*** | .41*** | - |  |  |
| 4. Stop task SSRT | -.39*** | -.45*** | -.25* | - |  |
| 5. Flanker Median RT in ms on  incongruent trials | .02 | -.01 | .10 | .50*** | - |
| 6. GNG False alarms in % | -.02 | -.10 | -.25* | .004 | -.15 |
| *Note*. BKE = Tic tac toe; COU = Mental counters; GNG = Go No Go task; incong. = incongruent; mean acc = mean accuracy; RT = Reaction Time; SSRT = Stop Signal Reaction Time.  **p*<.05 level; ***p*<.01 level; ****p*<.001 level. | | | | | |

| **Table S8**. Descriptives of tasks and questionnaires | | | | | | |
| --- | --- | --- | --- | --- | --- | --- |
|  | ADHD (*n* = 46) | | NC (*n* = 40) | | Total sample (*n* = 86) | |
| Variable | Mean (SD) | Range | Mean (SD) | Range | Mean (SD) | Range |
| Narrative outcome^a^ |  |  |  |  |  |  |
| Coherence | 3.98 (1.22) | 1-6 | 4.78 (1.10) | 2-6 | 4.35 (1.22) | 1-6 |
| Ambiguous ref. | 3.09 (0.37) | 0-11 | 1.58 (1.95) | 0-9 | 2.38 (2.36) | 0-11 |
| Causal language | 16.16 (11.45) | 0-43 | 16.78 (11.52) | 0-50 | 16.45 (11.42) | 0-50 |
| Irrelevant com. | 3.13 (3.31) | 0-11 | 3.60 (5.01) | 0-22 | 3.35 (4.18) | 0-22 |
| Misattributions | 2.02 (2.80) | 0-17 | 1.28 (1.34) | 0-6 | 1.67 (2.25) | 0-17 |
| Out of sequence | .18 (.49) | 0-2 | .10 (.30) | 0-1 | .14 (.41) | 0-2 |
| Mo-Sy errors | 3.36 (3.24) | 0-17 | 2.18 (2.41) | 0-12 | 2.80 (2.92) | 0-17 |
| MLU | 7.05 (1.58) | 3.65-11.50 | 7.48 (1.40) | 4.19-9.84 | 7.25 (1.50) | 3.65-11.5 |
| ISL | 3.91 (3.26) | 0-15 | 4.58 (4.76) | 0-24 | 4.22 (4.03) | 0-24 |
| CELF-4 Core language  summary score | 22.72 (6.97) | 7-37 | 30.13 (6.49) | 15-40 | 26.16 (7.67) | 7-40 |
| WMU^a^ |  |  |  |  |  |  |
| Tic tac toe mean acc. | .73 (.21) | .10-1.00 | .79 (.21) | .07-1.00 | .75 (.21) | 07-1.00 |
| Mental counter  mean acc. | .65 (.26) | .00-1.00 | .76 (.23) | .00-1.00 | .71 (.25) | .00-1.00 |
| Spatial 2-back  mean acc. | .61 (.18) | .25-.94 | .75 (.15) | .42-.96 | .67 (.18) | .25-.96 |
| IN^b^ |  |  |  |  |  |  |
| Flanker Median RT  on incong. trials | 668.09 (176.20) | 325.00-1027.50 | 615.39 (141.83) | 295.50-1044.50 | 642.08 (161.31) | 295.50- 1044.50 |
| Stop task SSRT | 323.91 (107.64) | 167.06-603.19 | 292.74 (86.79) | 176.79-539.47 | 307.90 (98.05) | 167.06-603.19 |
| GNG False alarms % | 14.19 (11.52) | 0-45 | 10.13 (10.28) | 0-50 | 12.08 (11.01) | 0-50 |
| *Note*. CELF-4 = Clinical Evaluation of Language Fundamentals-4; IN = Response Inhibition; incong. = incongruent; ISL = Internal state language; GNG = Go No Go task; mean ac. = mean accuracy; MLU = Mean Length Utterance; Mo-Sy = Morpho-syntactic; ref. = references; SD = Standard Deviation; SSRT = Stop Signal Reaction Time; WMU = Working Memory Updating. | | | | | | |
| ^a^ADHD *n* = 45; total sample *n* = 85; all WMU outcomes mean accuracy, converted to summary z-scores.  ^b^ADHD *n* = 41; PBC *n* = 39; total sample *n* = 80, converted to summary z-scores. | | | | | | |

# **Figure S2**.

# Multiple mediation model of the effect of working memory, inhibition and core language on the association between ADHD and coherence.

*Note.* NC = Neurotypical comparison children; WMU = Working Memory Updating.

****p*<.001

# **Figure S3**.

# Multiple mediation model of the effect of working memory, inhibition and core language on the association between ADHD and ambiguous references.

*Note.* NC = Neurotypical comparison children; WMU = Working Memory Updating.

****p*<.001

Table S9 Multivariate multiple regression model with eight narrative language outcomes as the dependent variables and diagnostic status, age, SES, and sex as the independent variables

| Narrative  aspect | Dependent variable | Independent variable | Unstand. | | | | Stand. | |
| --- | --- | --- | --- | --- | --- | --- | --- | --- |
|  |  |  | B | *S.E.* | *z* | *p* | *β^a^* | *R^2^* |
| Coherence | Coherence | Diagnostic status | -0.65 | -0.23 | -2.84 | .004 | -0.53 | .218*** |
|  |  | Age | 0.32 | 0.10 | 3.31 | .001 | 0.27 |  |
|  |  | Sex | 0.70 | 0.242 | 2.43 | .015 | 0.57 |  |
| Cohesion | Ambiguous references | SES  Diagnostic status | 0.01  1.83 | 0.05  0.44 | 0.25  4.20 | .805  <.001 | 0.01  0.77 | .141** |
|  |  | Age | -0.38 | 0.25 | -1.52 | .128 | -0.16 |  |
|  |  | Sex | -0.06 | 0.48 | -0.11 | .909 | -0.02 |  |
|  | Causal conjunctions | SES  Diagnostic status | 0.07  0.63 | 0.10  2.49 | 0.71  0.25 | .476  .800 | 0.03  0.05 | .027 |
|  |  | Age | -0.30 | 1.13 | -0.26 | .792 | -0.03 |  |
|  |  | Sex | -0.37 | 2.71 | -0.14 | .891 | -0.03 |  |
| Disruptions | Irrelevant comments | SES  Diagnostic status | 0.77  0.03 | 0.48  0.85 | 1.60  0.03 | .109  .976 | 0.07  0.01 | .014 |
|  |  | Age | 0.28 | 0.325 | 0.86 | .387 | 0.141 |  |
|  |  | Sex | 0.18 | 0.922 | 0.19 | .864 | -0.064 |  |
|  | Misattribution | SES  Diagnostic status | -0.08  0.85 | 0.16  0.40 | -0.49  2.11 | .626  .035 | -0.02  0.37 | .043 |
|  |  | Age | -0.01 | 0.18 | -0.03 | .980 | -0.002 |  |
|  |  | Sex | 0.37 | 0.48 | 0.79 | .574 | 0.16 |  |
| Syntactical complexity | Morpho-syntactic errors | SES  Diagnostic status | -0.08  1.35 | 0.14  0.59 | -0.57  2.32 | .572  .021 | -0.04  0.54 | .125* |
|  |  | Age | -0.62 | 0.26 | -2.38 | .017 | -0.25 |  |
|  |  | Sex | -0.17 | 0.67 | -0.25 | .799 | -0.07 |  |
|  | MLU | SES  Diagnostic status | 0.01  -0.39 | 0.11  0.34 | 0.10  -1.17 | .924  .240 | 0.004  -0.26 | .340*** |
|  |  | Age | 0.70 | 0.11 | 6.60 | <.001 | 0.47 |  |
|  |  | Sex | 0.70 | 0.31 | 2.23 | .026 | 0.47 |  |
| ISL | ISL | SES  Diagnostic status  Age  Sex | 0.15  0.29 | 0.06  0.83 | 2.41  0.35 | .016  .730 | 0.10  0.08 | .022 |
|  |  |  | 0.34 | 0.28 | 1.23 | .219 | 0.09 |  |
|  |  |  | 1.07 | 0.97 | 1.10 | .271 | 0.28 |  |
|  |  | SES | 0.05 | 0.13 | 0.39 | .699 | 0.01 |  |

# *Note*. ISL = Internal State Language, MLU = Mean Length Utterance, SE = Standard Error, SES = Social Economic Status.

# Table S10 Multiple mediation model of the effect of WMU, IN, and core language on the association between ADHD and ambiguous references (controlling for age, sex, and SES)

|  |  | |  | Unstand. | | | |  | |  | Stand. | |
| --- | --- | --- | --- | --- | --- | --- | --- | --- | --- | --- | --- | --- |
|  | *B* | | *SE* | *z* | | | *CI^a^* | | | *p* | *β ^b^* | *CI β^a^* |
|  | Effects of diagnostic status on morpho-syntactic errors (controlling for age and sex) | | | | | | | | | | | |
| Total effect | 1.84 | 0.47 | | | 3.92 | [1.04, 2.88] | | | <.001 | | 0.77 | [0.43, 1.11] |
| Total indirect | 0.46 | 0.39 | | | 1.18 | [-0.26, 1.18] | | | .238 | | 0.19 | [-0.13, 0.49] |
| Total direct | 1.39 | 0.56 | | | 2.48 | [0.26, 2.40] | | | .013 | | 0.58 | [0.08, 1.02] |
| Specific indirect effects  Mediator | | | | |  |  | | |  | |  |  |
| WMU | 0.04 | 0.32 | | | 0.13 | [-0.59, 0.69] | | | .893 | | 0.02 | [-0.29, 0.27] |
| IN | -0.12 | 0.18 | | | -0.70 | [-0.63, 0.10] | | | .485 | | -0.05 | [-0.26, 0.04] |
| Core lang. | 0.54 | 0.33 | | | 1.65 | [-1.26, 0.04] | | | .099 | | 0.22 | [-0.03, 0.52] |

*Note.* CI = bootstrapped confidence intervals, IN = response inhibition, lang. = language, SE = standard error, SES = social economic status, WMU = working memory updating.

^a^95% CIs.

^b^y-standardization.

# Table S11 Multiple mediation model of the effect of WMU, IN, and core language on the association between ADHD and narrative coherence (controlling for age, sex, and SES)

|  |  | Unstand. | | | |  |  | | Stand. | |
| --- | --- | --- | --- | --- | --- | --- | --- | --- | --- | --- |
|  | *B* | *SE* | | *z* | | *CI^a^* | *p* | | *β ^b^* | *CI β^a^* |
|  | Effects of diagnostic status on coherence (controlling for age and sex) | | | | | | | | | |
| Total effect | -0.65 | | 0.24 | -2.71 | [-1.12, -0.14] | | | .007 | -0.53 | [-0.85, -0.12] |
| Total indirect | -0.15 | | 0.20 | -0.75 | [-0.19, 0.62] | | | .454 | -0.12 | [-0.50, 0.16] |
| Total direct | -0.50 | | 0.32 | -1.59 | [-1.18, 1.06] | | | .113 | -0.41 | [-0.16, 0.88] |
| Specific indirect effects  Mediator | | | |  |  | | |  |  |  |
| WMU | -0.02 | | 0.17 | -0.10 | [-0.41, 0.28] | | | .921 | -0.01 | [-0.33, 0.24] |
| IN | -0.03 | | 0.07 | -0.36 | [-0.05, 0.32] | | | .723 | -0.02 | [-0.04, 0.23] |
| Core lang. | -0.14 | | 0.20 | -0.69 | [-0.24, 0.55] | | | .491 | -0.11 | [-0.20, 0.44] |

*Note.* CI = bootstrapped confidence intervals, IN = response inhibition, lang. = language, SE = standard error, SES = social economic status, WMU = working memory updating.

^a^95% CIs.

^b^y-standardization.

# Table S12 Multiple mediation model of the effect of WMU, IN, and core language on the association between ADHD and morphosyntactic errors (controlling for age, sex, and SES)

|  |  | | Unstand. | | | | | |  | Stand. | |
| --- | --- | --- | --- | --- | --- | --- | --- | --- | --- | --- | --- |
|  | *B* | | *SE* | | *z* | | *CI^a^* | | *p* | *β ^b^* | *CI β^a^* |
|  | Effects of diagnostic status on ambiguous references (controlling for age and sex) | | | | | | | | | | |
| Total effect | 1.37 | 0.62 | | 2.20 | | [0.28, 2.73] | | .028 | | 0.54 | [0.08, 1.03] |
| Total indirect | 0.58 | 0.44 | | 1.32 | | [-0.21, 1.53] | | .188 | | 0.23 | [-0.10, 0.57] |
| Total direct | .79 | 0.72 | | 1.09 | | [-0.59, 2.22] | | .276 | | 0.31 | [-0.26, 0.88] |
| Specific indirect effects  Mediator | | | |  | |  | |  | |  |  |
| WMU | 0.04 | 0.30 | | 0.15 | | [-0.53, 0.75] | | .884 | | 0.02 | [-0.22, 0.29] |
| IN | -0.15 | 0.26 | | -0.58 | | [-0.86, 0.23] | | .562 | | -0.06 | [-0.31, 0.10] |
| Core lang. | 0.68 | 0.33 | | 2.08 | | [0.13, 1.43] | | .038 | | 0.27 | [0.05, 0.56] |

*Note.* CI = bootstrapped confidence intervals, IN = response inhibition, lang. = language, SE = standard error, SES = social economic status, WMU = working memory updating.

^a^95% CIs.

^b^y-standardization.

# References

APA, A. P. A. (2013). *Diagnostic and Statistical Manual of Mental Disorders, 5th Edition.* (A. P. Association, Ed.). American Psychiatric Association. <https://doi.org/doi:10.1176/9780890425596>

Baixauli Fortea, I., Berenguer Forner, C., Colomer, C., Casas, A. M., & Miranda, B. R. (2018). “Communicative skills in Spanish children with Autism Spectrum Disorder and children with Attention Deficit Hyperactivity Disorder. Analysis through parents’ perceptions and narrative production” [Article]. *Research in Autism Spectrum Disorders*, *50*, 22-31. <https://doi.org/10.1016/j.rasd.2018.02.006>

Baixauli, I., Colomer, C., Rosello, B., & Miranda, A. (2016). Narratives of children with high-functioning autism spectrum disorder: A meta-analysis. *Research in Developmental Disabilities*, *59*, 234-254. <https://doi.org/10.1016/j.ridd.2016.09.007>

Flory, K., Milich, R., Lorch, E. P., Hayden, A. N., Strange, C., & Welsh, R. (2006). Online Story Comprehension among Children with ADHD: Which Core Deficits are Involved? *Journal of Abnormal Child Psychology*, *34*(6), 853-865. <https://doi.org/http://dx.doi.org/10.1007/s10802-006-9070-7>

Friedman, P. D., Melnick, G., Jiang, L., & Hamilton, Z. (2008). Violent and disruptive behavior among drug-involved prisoners: Relationship with psychiatric symptoms. *Behavioral Sciences & the Law*, *26*(4), 389-401. <https://doi.org/http://dx.doi.org/10.1002/bsl.824>

Huizinga, M., Dolan, C. V., & van der Molen, M. W. (2006). Age-related change in executive function: developmental trends and a latent variable analysis. *Neuropsychologia*, *44*(11), 2017-2036. <https://doi.org/10.1016/j.neuropsychologia.2006.01.010>

Huyser, C., Veltman, D. J., Wolters, L. H., de Haan, E., & Boer, F. (2011). Developmental aspects of error and high-conflict-related brain activity in pediatric obsessive-compulsive disorder: a fMRI study with a Flanker task before and after CBT. *J Child Psychol Psychiatry*, *52*(12), 1251-1260. <https://doi.org/10.1111/j.1469-7610.2011.02439.x>

Kuijper, S. J. M., Hartman, C. A., Bogaerds-Hazenberg, S. T. M., & Hendriks, P. (2017). Narrative production in children with autism spectrum disorder (ASD) and children with attention-deficit/hyperactivity disorder (ADHD): Similarities and differences. *Journal of abnormal psychology (1965)*, *126*(1), 63-75. <https://doi.org/10.1037/abn0000231>

Liles, B. Z., & Purcell, S. (1987). Departures in the spoken narratives of normal and language-disordered children. *Applied Psycholinguistics*, *8*(2), 185-202. <https://doi.org/10.1017/S0142716400000205>

Logan, G. D. (1994). On the ability to inhibit thought and action: A users' guide to the stop signal paradigm. In (pp. 189).

Logan, G. D., Logan, G. D., & Schachar. (1997). Impulsivity and Inhibitory Control. *Psychological science*, *8*(1), 60-64. <https://doi.org/10.1111/j.1467-9280.1997.tb00545.x>

Norbury, C. F., & Bishop, D. V. M. (2003). Narrative skills of children with communication impairments. *International Journal of Language & Communication Disorders*, *38*(3), 287-313. <https://doi.org/http://dx.doi.org/10.1080/136820310000108133>

Potratz, J. R., Gildersleeve-Neumann, C., & Redford, M. A. (2022). Measurement Properties of Mean Length of Utterance in School-Age Children. *Lang Speech Hear Serv Sch*, *53*(4), 1088-1100. <https://doi.org/10.1044/2022_lshss-21-00115>

Raven, J. (2000). The Raven's progressive matrices: change and stability over culture and time. *Cogn Psychol*, *41*(1), 1-48. <https://doi.org/10.1006/cogp.1999.0735>

Rumpf, A.-L., Kamp-Becker, I., Becker, K., & Kauschke, C. (2012). Narrative competence and internal state language of children with Asperger Syndrome and ADHD. *Research in Developmental Disabilities*, *33*(5), 1395-1407. <https://doi.org/http://dx.doi.org/10.1016/j.ridd.2012.03.007>

Semel, E. M., Wiig, E. H., & Secord, W. (2003). CELF 4 : Clinical evaluation of Language fundamentals 4. In (Danish version ed.). Toronto, Canada: The Psychological Corporation/A Harcourt Assessment Company.

Siller, M., Swanson, M. R., Serlin, G., & Teachworth, A. G. (2014). Internal state language in the storybook narratives of children with and without autism spectrum disorder: Investigating relations to theory of mind abilities. *Research in Autism Spectrum Disorders*, *8*(5), 589-596. <https://doi.org/http://dx.doi.org/10.1016/j.rasd.2014.02.002>

Snyder, H. R., Miyake, A., & Hankin, B. L. (2015). Advancing understanding of executive function impairments and psychopathology: Bridging the gap between clinical and cognitive approaches. *Frontiers in psychology*, *6*.

Swanson, J. M., Schuck, S., Porter, M. M., Carlson, C., Hartman, C. A., Sergeant, J. A., Clevenger, W., Wasdell, M., McCleary, R., Lakes, K., & Wigal, T. (2012). Categorical and Dimensional Definitions and Evaluations of Symptoms of ADHD: History of the SNAP and the SWAN Rating Scales. *Int J Educ Psychol Assess*, *10*(1), 51-70.

Tannock, R., Purvis, K. L., & Schachar, R. J. (1993). Narrative abilities in children with attention deficit hyperactivity disorder and normal peers. *Journal of Abnormal Child Psychology*, *21*(1), 103-117. <https://doi.org/http://dx.doi.org/10.1007/BF00910492>

Tsujimoto, S., Kuwajima, M., & Sawaguchi, T. (2007). Developmental fractionation of working memory and response inhibition during childhood. *Exp Psychol*, *54*(1), 30-37. <https://doi.org/10.1027/1618-3169.54.1.30>

Wechsler, D. (2003). *Wechsler Intelligence Scale for Children, Fourth Edition (WISC-IV) administration and scoring manual*. Pearson.

Wechsler, D. (2014). *Wechsler Intelligence Scale for Children, Fifth Edition (WISC​-V): Technical and Interpretive Manual​*. Pearson.

Williams, B. R., Ponesse, J. S., Schachar, R. J., Logan, G. D., & Tannock, R. (1999). Development of inhibitory control across the life span. *Developmental Psychology*, *35*(1), 205-213. <https://doi.org/10.1037/0012-1649.35.1.205>

Young, M. E., Sutherland, S. C., & McCoy, A. W. (2018). Optimal go/no-go ratios to maximize false alarms. *Behavior Research Methods*, *50*(3), 1020-1029. <https://doi.org/10.3758/s13428-017-0923-5>
